# Enhanced photoelectrochemical and photocatalytic activity of WO_3_-surface modified TiO_2_ thin film

**DOI:** 10.1186/s11671-015-0745-2

**Published:** 2015-02-06

**Authors:** Mohammad Qamar, Qasem Drmosh, Muhammad I Ahmed, Muhammad Qamaruddin, Zain H Yamani

**Affiliations:** Center of Excellence in Nanotechnology (CENT), King Fahd University of Petroleum and Minerals (KFUPM), Box 498, Dhahran, 31261 Saudi Arabia

**Keywords:** Photocatalysis, WO_3_/TiO_2_ thin film, Photoelectrochemistry, Sputtering

## Abstract

Development of nanostructured photocatalysts for harnessing solar energy in energy-efficient and environmentally benign way remains an important area of research. Pure and WO_3_-surface modified thin films of TiO_2_ were prepared by magnetron sputtering on indium tin oxide glass, and photoelectrochemical and photocatalytic activities of these films were studied. TiO_2_ particles were <50 nm, while deposited WO_3_ particles were <20 nm in size. An enhancement in the photocurrent was observed when the TiO_2_ surface was modified WO_3_ nanoparticles. Effect of potential, WO_3_ amount, and radiations of different wavelengths on the photoelectrochemical activity of TiO_2_ electrodes was investigated. Photocatalytic activity of TiO_2_ and WO_3_-modified TiO_2_ for the decolorization of methyl orange was tested.

**Graphical abstract:**
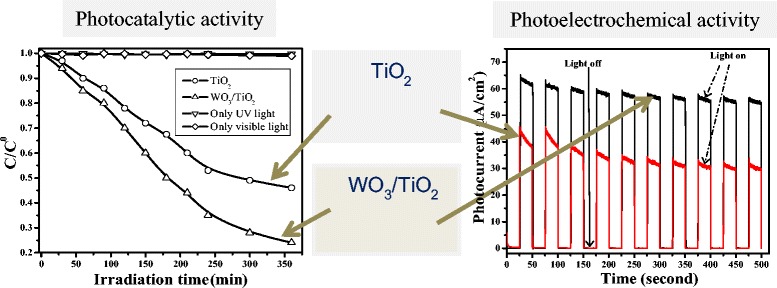
WO_3_-surface modified TiO_2_ film showing better photocatalytic and photoelectrocatalytic activity.

WO_3_-surface modified TiO_2_ film showing better photocatalytic and photoelectrocatalytic activity.

## Background

Semiconductor-mediated photocatalytic process (advanced oxidation process (AOT)) has emerged as one of the most promising chemical oxidation processes, anticipated to play a crucial role in water treatment as standalone processes or in combination with conventional technologies [1-4]. It has now been well established that metal oxide-mediated photocatalysis is an attractive and promising technology to be applied in environmental clean up, clean energy production (H_2_ production from water splitting), self-cleaning surface, CO_2_ reduction under solar light or illuminated light source, and green synthetic organic chemistry (some selective photocatalytic oxidation reactions) [5-12]. The fundamentals of heterogeneous photocatalytic oxidation processes have been well documented in the literature [13-15]. Briefly, by shining light of energy equal to or greater than the band gap of semiconductor, an electron may be promoted from the valence band to the conduction band (e^−^cb) leaving behind an electron vacancy or ‘hole’ in the valence band (h^+^vb). If charge separation is maintained, the electron and hole may migrate to the catalyst surface where they participate in redox reactions with absorbed species. Specially, h^+^vb may react with surface-bound H_2_O or OH^−^ to produce hydroxyl radical (OH^•^), and e^−^cb is picked up by oxygen to generate superoxide radical anion (O_2_^−•^). These reactive species are primarily responsible for the photodegradation of organic pollutants. TiO_2_ is currently the best known and most widely used photocatalytic material because it is photostable, nontoxic, and relatively inexpensive [3,4]. One practical problem associated with semiconductors is the undesired electron/hole recombination process which in the absence of a proper electron acceptor or donor is extremely efficient and hence represents the major energy-wasting step, thus limiting the quantum yield (more than 90% charge carriers recombine). Several scientific strategies, such as doping with transition metal ions [16], deposition of noble metals [17], dye sensitization [18], and coupling with other low band gap semiconductors [19-21], have been put forward to prevent the electron-hole pair recombination in the semiconductor and improve the photocatalytic activity. Among the oxide semiconductors, coupling TiO_2_ with WO_3_ has been the subject of intensive investigations owing to a small band gap between 2.4 and 2.8 eV, a deeper valence band (+3.1 eV), effective absorption of the solar spectrum, unique physicochemical properties, and resilience to photocorrosion [22-25].

Semiconductor-based thin films are the subject of great interest not only for their excellent properties such as high chemical inertness, high thermal stability, and corrosion resistance but also for their excellent mechanical, optical, electrical, electronic, and catalytic properties. To the best of our knowledge, no attempt has been made to study the photoelectrochemical and photochemical property of WO_3_/TiO_2_ bilayers prepared by sputtering method. In the study presented here, we demonstrated the preparation of pure and WO_3_-surface modified TiO_2_ thin films using plasma-assisted sputtering method and studied their photoelectrochemical and photocatalytic properties.

## Methods

### Materials

Titanium (99.999%) and tungsten (99.99%) targets were obtained from Semiconductor Wafer, Inc. (Hsinchu, Taiwan), while indium tin oxide (ITO)-coated glass slide, sodium sulfate (>99.0%), and methyl orange (dye content ca 85%) were obtained from Sigma-Aldrich (St. Louis, MO, USA).

### Preparation of thin films

Thin films were fabricated by automatic sputter coater (NSC-4000) onto ITO substrates using high-purity titanium and tungsten targets. Before sputtering, the substrates were cleaned for 15 min in methanol by ultrasonication. Furthermore, surfaces of titanium and tungsten targets were cleaned before each experiment by a pre-sputtering process for 1 min. The base pressure in the chamber was less than 2 × 10^−6^ torr, and the working pressure was set to 7 mtorr by adjusting the O_2_ gas flow at 70 sccm. The distance between the target and the substrate was fixed at 10 cm. The depositions of thin films were done at two different steps and conditions: TiO_2_ thin film was deposited first using 120 W for 40 min time deposition using radio frequency (rf) in pure oxygen. In the second step, WO_3_ flashed using a DC reactive sputtering with 70 W for 1, 2.5, 5, and 10 min on TiO_2_ thin film in high-purity oxygen environment. The substrate temperature was 300 K, while the sputtering rates of titanium and tungsten were 0.7 and 1.5 Å/s, respectively.

### Characterization

Morphological characterization of the films was carried out by employing field emission scanning electron microscope (FESEM) while the elemental analysis was performed by energy-dispersive X-ray spectroscopy (EDS). Optical property was studied using a UV-vis spectrophotometer.

### Evaluation of photoelectrochemical and photocatalytic activity

Photoelectrochemical behavior was studied using a three-electrode photoelectrochemical cell and a potentiostat. Saturated calomel electrode (SCE) and coiled platinum electrodes were used as a reference and counter electrodes, respectively. Irradiation of films was carried out under light with different wavelengths generated from 300-W xenon lamp. In all cases, the coated side (consisting of TiO_2_ or WO_3_/TiO_2_) of the films was irradiated. Different modules for UV and UV-vis along with cut off filters were used to get the radiations of desired wavelengths.

The photocatalytic tests were performed in a small photocell equipped with a quartz window and a magnetic stirring bar. For irradiation experiments, 100 mL of methyl orange solution with desired concentration was taken into the photocell and thin film slide was immersed into the dye solution. Irradiation was carried out using the abovementioned xenon light source. Samples (approximately 5 mL) were taken at regular time intervals from the cell, and concentration was determined by UV-visible spectrophotometer. Decomposition (decrease in absorption intensity vs. irradiation time) of the dye was monitored by measuring the change in absorbance.

## Results and discussion

**Figure 1:**
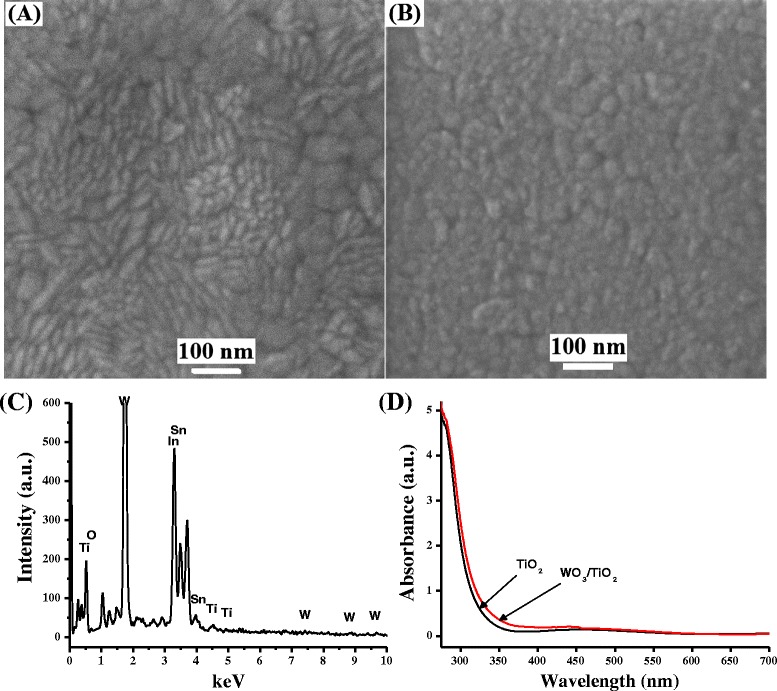
**FESEM images, EDS spectrum, and absorption spectra of TiO**_**2**_ **and WO**_**3**_ **/TiO**_**2**_ **bilayer films.** Field emission scanning electron microscopic image of **(A)** TiO_2_ film and **(B)** WO_3_/TiO_2_ bilayer film (deposition time = 5 min), **(C)** EDS spectrum of WO_3_/TiO_2_ bilayer film, and **(D)** absorption spectra of TiO_2_ and WO_3_/TiO_2_ films (deposition time = 5 min).

Structural analyses of films were carried out using FESEM, and representative images are illustrated in Figure 1. Pure titania film (Figure 1A) was found to be composed of nonspherical and irregular grains of <50-nm size with a certain degree of concavities between the grains. On the other hand, WO_3_ particles were somewhat spherical in shape and smaller in size (<20 nm) with homogeneous and narrow distribution throughout the surface of TiO_2_, as illustrated in Figure 1B. A representative EDS spectrum of film has been delineated in Figure 1C which indicated the presence of W, Ti, and O, along with Sn and In which are coming from ITO coatings. Unlabeled peaks may be ascribed to various elements present in glass slide.

Figure 1D shows the UV-visible absorption spectra of pure TiO_2_ as well as TiO_2_ films modified with WO_3_. The spectra corresponded to a typical absorption behavior of TiO_2_, as obtained by other researchers as well. The absorption spectra of WO_3_-modified titania films were almost similar to that of bare titania, and surface modification by a low band gap semiconductor (WO_3_, approximately 2.8 eV) could not contribute in any notable visible band gap narrowing. The band gaps of bare TiO_2_ film and WO_3_/TiO_2_ film, prepared after 5 min, were calculated to be 3.3 and 3.2 eV, respectively.

**Figure 2:**
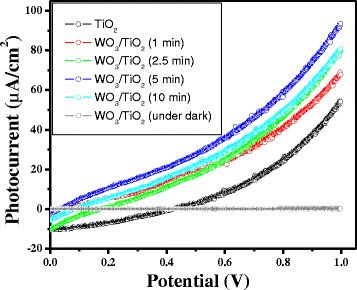
**Potentiodynamic behavior of TiO**_**2**_ **with respect to the thickness of WO**_**3**_ **layers.**

Coated side consisting of TiO_2_ or WO_3_/TiO_2_ of the electrodes was illuminated with UV light radiation, and obtained potentiodynamic behavior is shown in Figure 2. It is obvious from the figure that the photocurrent was increased with increasing the applied voltage. Figure 2 also demonstrates the effect of the thickness of WO_3_, which was deposited on TiO_2_ surface and controlled by deposition time, on the potentiodynamic response of electrodes. Using thickness monitoring, the thickness of TiO_2_ film was calculated to approximately 170 nm, whereas the thickness of deposited WO_3_ layers was calculated to be about 10, 20, 45, and 90 nm for 1, 2.5, 5.0, and 10 min, respectively. It is interesting to note that the photocurrent density was found to increase with the increase in WO_3_ amount up to a critical amount, and a further increase failed to contribute positively on the overall photocurrent efficiency of the electrode. The overall effect of WO_3_ amounts on TiO_2_ surface was more significant at or higher than 0.6 V. This behavior may be explained in terms of interfacial electron transfer between WO_3_ and TiO_2_ as well as the extent of TiO_2_ surface coverage by WO_3_. In case of partial or optimum coverage of the TiO_2_ surface by WO_3_ particles, both oxide surfaces may absorb incident photons and generate charge carriers, which may undergo an interfacial charge transfer process thereby enhancing the overall flow of current. On the other hand, when the coverage of TiO_2_ surface by WO_3_ exceeds the critical limit or TiO_2_ surface is mostly covered by WO_3_, photons will largely be absorbed by WO_3_ ensuing lesser generation of electrons or photocurrents, noting the fact that WO_3_ is an intrinsically less active photocatalyst than TiO_2_.

**Figure 3:**
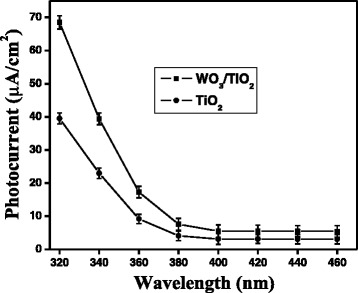
**Action spectra of photocurrent in presence of TiO**_**2**_ **and WO**_**3**_ **/TiO**_**2**_ **(deposition time = 5 min) thin film electrodes.**

**Figure 4:**
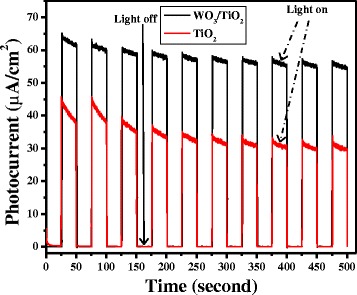
**Voltammograms under intermittent illumination/darkness of TiO**_**2**_ **and WO**_**3**_ **/TiO**_**2**_ **thin films (deposition time = 5 min).**

Photocurrent action spectra or effect of wavelength on the photocurrent of pure and WO_3_-modified TiO_2_ electrodes (deposition time = 5 min) was measured at a constant applied anodic potential (0.9 V). Furthermore, three scans were performed at each wavelength and results together with error bars are presented in Figure 3. Since the linear relationship between light intensity and photocurrent can interfere with the effect of wavelengths and lead to an elusive conclusion, the intensity of incident light was kept constant, with the help of neutral density filters equipped with the xenon light source, at different wavelengths. Both the films showed maximum photocurrent at 320 nm and found to decrease continuously with the increase in wavelength (or decrease in photonic energy) up to 460 nm. However, the photocurrent of bare TiO_2_ was significantly less than that of WO_3_-modified TiO_2_ at any wavelength studied. By keeping in mind that the illumination of catalyst’s surface with high-energy photons may induce some changes by creating surface states [26], the direction of wavelength scanning was followed from higher to lower, i.e., from low-energy photons to high-energy photons in order to minimize any such kind of effect. The trend of photocurrent flow as a function of wavelengths agreed fairly well with absorption spectra of samples (Figure 3). Furthermore, since the specimens may show a certain degree of current drift over time scales of 5 to 10 min, ambiguity between photocurrents under illumination and dark current is created. Photocurrents (under illumination and dark current) were measured in a single experiment by turning the light on and off after every 25 s for more than 8 min at a constant applied voltage 0.9 V, and obtained results are presented in Figure 4. The photocurrent generated instantaneously upon illumination and reached a steady state while no current was observed under dark, even at high applied potential (0.9 V). It could be seen from the figure that both the electrodes possessed similar and reasonably good stability under studied experimental conditions and WO_3_-modified TiO_2_ electrode showed better photocurrent.

**Figure 5:**
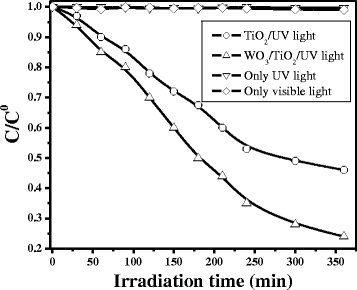
**Change in concentration of methyl orange aqueous solution with TiO**_**2**_ **/UV, WO**_**3**_ **/TIO**_**2**_ **/UV (deposition time = 5 min), UV, and visible light.**

Since the photocatalytic property of mixed-oxide thin films is also of great significance, the activity of bare TiO_2_ and WO_3_-modified TiO_2_ was evaluated by studying the photooxidation of a dye, namely methyl orange, without applying any potential under ultraviolet radiation, and obtained results are illustrated in Figure 5. Thin film of TiO_2_ modified with WO_3_ (deposition time = 5 min) showed approximately 40% more activity for dye decolorization than that of pure TiO_2_. Photocatalytic experiments were also carried out under dark, without any applied potential and in the absence of photocatalyst, but no change in dye concentration was observed which indicates that the dye decolorization processes was truly photocatalytic.

**Figure 6:**
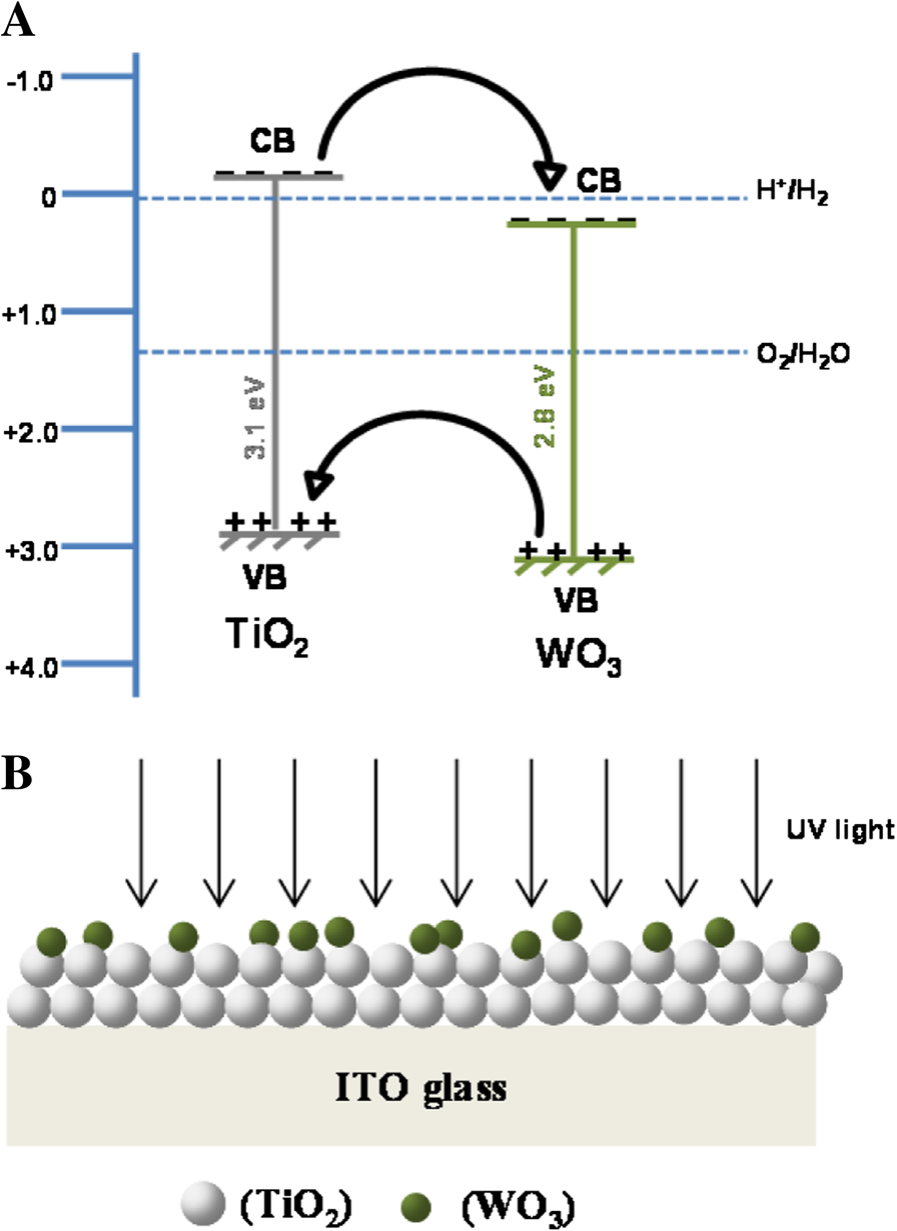
**Schematic showing (A) the interfacial charge transfer between WO**_**3**_ **and TiO**_**2**_ **and (B) the direction of UV light.**

In summary, a significant improvement in the aforementioned photoelectrochemical and photocatalytic processes of TiO_2_ was observed after surface modification with highly dispersed and very small size of WO_3_ nanoparticles. This effect could be attributed to the basic mechanism of photocatalysis. A significant amount of electrons and hole pairs could be generated upon illumination of catalyst’s surface with photons of appropriate energy. Unfortunately, most of the excited charge carriers (approximately 95%) go through the recombination process, in the absence of an effective electron transfer mechanism, which is a major energy-wasting process and overrides limitation of photocatalysts. When WO_3_ is effectively coupled with TiO_2_, the excited charge carriers undergo interfacial charge transfer phenomena and thus experience extended lifetime which in turn improved the photoelectrochemical and photocatalytic process. A plausible mechanism, based on the energy band diagram, showing the interfacial charge transfer between TiO_2_ and WO_3_ under UV light and applied anodic potential is presented in Figure 6A, together with schematic of WO_3_/TiO_2_/ITO layered structure (Figure 6B) [27].

## Conclusions

The surface of TiO_2_ could be effectively functionalized by depositing nanoparticulates of WO_3_ by magnetron sputtering method. Microscopic analyses showed that TiO_2_ nanoparticles were nonspherical, irregular in shape, and <50 nm in size while WO_3_ nanoparticles were well distributed throughout TiO_2_ surface and were smaller (<20 nm) in size as compared to TiO_2_. Improvement in photocurrent and photocatalytic activity of TiO_2_ thin film was observed, approximately 50% and approximately 40%, respectively, after surface modification with WO_3_ nanoparticles. Both the films, TiO_2_ and WO_3_/TiO_2_, showed the highest photocurrent under 320-nm radiation, which continuously decreased with decreasing photonic energy. This study has presented a representative example by investigating the photoelectrochemical and the photochemical behavior of WO_3_-modified TiO_2_ thin film; this simple and effective methodology could be applied to develop other mixed-metal oxides for solar energy conversion and environmental decontamination.
